# Ultrasensitive interferons quantification reveals different cytokine profile secretion in inflammatory myopathies and can serve as biomarkers of activity in dermatomyositis

**DOI:** 10.3389/fimmu.2025.1529582

**Published:** 2025-02-12

**Authors:** Loïs Bolko, Céline Anquetil, Alba Llibre, Solène Maillard, Damien Amelin, Karim Dorgham, Vincent Bondet, Océane Landon-Cardinal, Ségolène Toquet, Kuberaka Mariampillai, Samuel Malatre, Alexandrine Mahoudeau, Baptiste Hervier, Mathieu Rodero, Guy Gorochov, Darragh Duffy, Olivier Benveniste, Yves Allenbach

**Affiliations:** ^1^ Rheumatology, Maison Blanche Hospital, Reims, France; ^2^ Department of Internal Medicine and Clinical Immunology, National Reference Center for Rare NeuroMuscular Disorders, Pitié-Salpêtrière Hospital, Paris, France; ^3^ Sorbonne Université, Institut National de la Santé et de la Recherche Médicale (INSERM), Association Institut de Myologie, Center of Research in Myology, UMRS, Paris, France; ^4^ Translational Immunology Unit, Institut Pasteur, Université Paris Cité, Paris, France; ^5^ Sorbonne Université, Institut National de la Santé et de la Recherche Médicale (INSERM), Centre d’Immunologie et des Maladies Infectieuses (CIMI-Paris), Assistance Publique Hôpitaux de Paris (AP-HP), Hôpital Pitié-Salpêtrière, Paris, France; ^6^ Department of Medicine, University of Montreal, Montreal, QC, Canada; ^7^ Division of Rheumatology, Centre Hospitalier de l’Université de Montréal (CHUM), Montreal, QC, Canada; ^8^ Laboratory of Pharmacological and Toxicological Chemistry and Biochemistry, Centre National de la Recherche Scientifique (CNRS), Paris Cité University, Paris, France

**Keywords:** interferon, dermatomyositis, immune mediated necrotizing myopathie, Anti-synthetase syndrom, inclusion body myositis, biomarker

## Abstract

**Objective:**

The objective of this study was to evaluate the presence of different types of interferon in idiopathic inflammatory myopathies (IIM) and their subgroups using ultrasensitive cytokine detection techniques (SIMOA) and to assess their potential as activity biomarkers.

**Methods:**

Disease activity was measured at the time of serum collection and assessed by manual muscle testing eight (MMT8 score 0-150), muscle enzymes to calculate the Physician Global Assessment (PGA) (0-10). Patients were classified as active if PGA>5.Serum IFN-α and IFN-γ levels was measured using the single molecule array (SIMOA) technique. Serum IFN-β level was measured by Elisa. Correlation between IFN levels and disease activity were performed.

**Results:**

We included 242 IIM patients and found a good correlation between type I Interferon (IFN) and dermatomyositis disease activity. IFN-α and IFN-β was highly correlated with disease activity (r=0.76 and r=0,58). To evaluate whether the different types of Interferons could serve as biomarkers of activity, we generated ROC curves. Patients with active DM had a higher median IFN-α level (0.49 pg/ml [0.1-3.7]) compared with non-active patients (0.03 pg/ml [0.01-0.07] p<0.05). The area under the curve was 0.90 IC95 (0.76-0.97) p<0.05. Furthermore, Myositis-specific antibodies appear to be associated with a different secretion profile; patients with anti-MDA 5 antibodies had higher level of IFN-α than most other antibodies (6.58 vs 0.14 p<0.005). NXP2 had higher IFN-β level than patients with Tif1γ antibodies.

**Conclusion:**

Serum IFN-α level measured by SIMOA is a reliable biomarker of DM activity. Myositis-specific antibodies appear to be associated with a different secretion profile. This data needs to be confirmed in order to select the good therapeutics strategies in DM.

## 1 Introduction

Idiopathic inflammatory myopathies (IIM) are a heterogenous group of autoimmune diseases including four main groups: dermatomyositis (DM), anti-synthetase syndrome (ASyS), immune-mediated necrotizing myopathies (IMNM), and inclusion body myositis (IBM) (1, 2). IIM may manifest as a muscle-specific autoimmune disorder (IBM and IMNM) or as a systemic condition primarily affecting the skin, joints, and/or lungs (DM and ASyS).

The complexity of disease activity assessment in IIM arises from its heterogeneous nature. A core set of disease activity measures, aimed at evaluating improvement through a total improvement score calculated from two time points, has been proposed (3). However, reliable biomarkers are still required to assess disease activity at a single time point. While creatine kinase (CK) levels, one of the core set measures, are well correlated with disease activity in IMNM (4), they may lack sensitivity in patients with ASyS or DM (5).

Interferons (IFNs) play a significant role in the pathophysiology of IIM (6–8). There are three main types of IFN. Type I IFN are mainly represented by 13 subtypes of IFN-α and IFN-β. Type II IFN is only represented by IFN-γ and signal through a distinct receptor. Due to the low circulating levels of these cytokines, the IFN signature—an overexpression of IFN-stimulated genes —is typically used as an indirect measure, rather than direct IFN quantification. This signature has been identified in blood (9), muscle (7) and skin (10) of DM patients, and is associated with disease activity (8, 11). Type II IFN is related to CD8+ T cells and has been involved in ASyS and IBM (12, 13). There appears to be a type II IFN signature in IMNM muscle biopsies (14), but the data are still uncertain.

Despite the identification of IFN signatures in various tissues, results have been inconsistent, particularly with respect to the different subtypes of myositis (15, 16). No studies have evaluated the precise quantification of various cytokines along the IFN pathway in the different myositis subgroups. Recent advances in ultrasensitive technology have enabled the detection of very low concentrations of proteins, such as IFN-α and IFN-γ, at femtomolar levels (17), making it possible to measure these cytokines with greater accuracy.

The aim of this study was to assess the potential of type I and II IFN, using an ultrasensitive digital ELISA technology, as a blood biomarker of activity for IIM.

## 2 Patients and methods

### 2.1 Patients and sera

Patients were prospectively enrolled between 2011 and 2018 for the first cohort, in a tertiary center of IIM (Pitié-Salpêtrière Hospital, Paris, France).

A validation cohort was established for the dermatomyositis and anti-synthetase syndrome subgroups in an independent cohort of patients sampled between 2018 and 2023. They fulfilled the American college of rheumatology/European league against rheumatism (ACR/EULAR) classification criteria for myositis (18). Patients were classified into four categories: IBM [Lloyd’s criteria (19)], IMNM [ENMC 2017 (20)], ASyS in presence of anti-synthetase antibody and according to ENMC criteria (21) and DM (1, 22)

Sera were collected at diagnosis and/or during the follow-up and were rapidly (<3h) frozen after one centrifugation. All the sera were thawed only once to avoid potential freeze/thaw effects. Patients who had increased dose of corticosteroids (>0.5mg/kg and/or pulses) the week before the sampling were excluded as it may rapidly abrogate the IFN levels (23). Moreover, patients with active infectious diseases (e.g. flue or viral B hepatitis) were excluded. Thirty-three age-matched healthy donors (HD) from a French blood bank were used as negative controls.

### 2.2 Disease activity assessment

Using International Myositis Assessment and Clinical Studies Group core set measures the following assessments were performed: Manual Muscle Testing 8 (MMT8), Creatine Kinase (CK) level for the muscle domain and for extra-muscular domains, we used Myositis Disease Activity Assessment Tool (MDAAT) scoring [0-10] the extramuscular manifestation (3). Finally, to assess the global disease activity we used the Physician Global Activity (PGA) (3). Disease activity was assessed at the time of blood collection and the result was represented in a numeric scale (from 0 to 10; 0 corresponding to the remission without treatment and 10 the maximum disease activity).

### 2.3 IFN serum measurement

IFN-α and IFN-γ serum concentrations were measured using the high sensitivity Single Molecule Array (Simoa^®^) technology (Digital ELISA technology) (Quanterix SimoaTM IFN-α Reagent Kit, Lexington, MA, USA and Quanterix SimoaTM IFN-γ Reagent Kit, Lexington, MA, USA) according to the manufacturer protocols and as previously reported (9, 11, 17–19, 24, 25). Briefly, we collected 5 ml of blood from each patient and 400µl of serum per well was required for each SIMOA analysis.

The limit of detection (LOD) was 0.0035 pg/ml for IFN-α and 0.026 pg/ml for IFN-γ. The positivity threshold was defined as the mean plus three times the standard deviation of the 33 healthy donors (HD) and was 0.22 pg/ml for IFN-α and 1.97 pg/ml for IFN-γ.

For IFN-β serum quantification, Elisa test was used (PBL Assay Science, Piscataway, NJ, USA). The LOD was calculated by the mean value of the blank plus two times the standard deviation (positivity at 95% confidence) calculated on logarithmic values and was 1.24 pg/ml and the positivity threshold was defined by the mean plus three times the standard deviation of the HD and was 2.50 pg/ml.

### 2.4 Myositis specific antibody detection

The screening for Myositis-Specific Antibody (MSA) was performed with different line blot commercial assays as previously reported (1) using Euroimmun^®^ or Dteck^®^ immunoassays including anti-melanoma differentiation-associated protein 5 (anti-MDA5), -transcription intermediary factor-γ (anti-Tif1γ), -complex nucleosome remodeling histone deacetylase (anti-Mi2), -nuclear matrix protein 2 (anti-NXP2) and -SUMO-activating enzyme subunit 1 (anti-SAE1) for DM; -histidyl-ARNt synthetase (anti-Jo1), -threonine-ARNt synthetase (anti-PL7), -alanine-ARNt synthetase and -glycine-ARNt synthetase (anti-EJ) for ASyS; -signal recognition particle (anti-SRP) and -3-hydroxy-3-methylglutaryl-coenzyme A reductase (anti-HMGCR) for IMNM.

### 2.5 Statistical analysis

Quantitative variables were expressed as median with inter-quartile range, and numbers with proportions for categorical variables. Multiple comparisons were performed using Kruskal-Wallis test then Dunn’s *post-hoc* test for quantitative data. To analyse the correlation between IFN and disease activity assessed by the PGA, we performed Spearman’s rank correlation tests using Graphpad Prism 10^®^.

Positive threshold to discriminate active from inactive patient was assessed by ROC curve analysis. We used the Youden indice to minimise both the number of false-positive and false-negative results. CK and IFN values were transformed through a base-10 logarithm for analysis.

After verifying the absence of multicollinearity, we included IFN-α, IFN-γ and CK levels in binary multivariate logistic regression to determine the association with disease activity (binary outcome using PGA>5 to define active patients). P<0.05 was considered statistically significant.

### 2.6 Ethical

Written informed consent from each study patient and approval by local Ethics Committee (CPP Ile De France VI (2013-12-19), CCTIRS (N°14.323) and CNIL (AR158656)) were obtained.

## 3 Results

### 3.1 Patients’ characteristics

One hundred fifty-two patients were included in the first cohort (DM, n=50; ASyS, n=46; IMNM, n=32 and IBM, n=24) compared with 33 healthy donors. Main patients’ characteristics are shown in **Table 1**.

**Table 1:** Patients characteristics in the first cohort at time of blood sampling.

Diagnosis	DM	ASyS	IMNM	IBM	Total
n	50	46	32	24	222
Age (year)	53.2 ± 15.4	48.6 ± 14.5	49.6 ± 19.1	69.3 ± 8.3	53.6 ± 16.6
MSA, n (%)	35 (70)	46 (100)	32 (100)	12 (100)	125
MAA, n (%)	13 (26)	37 (80)	9 (28)	3 (13)	62
MMT8 (0-150)	142 [126-150]	150 [132-150]	132 [115-146]	120 [94-133]	138 [119-150]
CK level (UI/ml)	112 [60-460]	550 [123-1500]	780 [249-1332]	586 [296-1123]	432 [109-974]
MDAAT (0-60)	10 [3.5-17]	9 [2-21.5]	na	na	10 [2.5-17]
PGA (0-10)	5 [2-8]	5 [2-8]	5 [2-7]	na	5 [2-8]
Corticosteroids n (%)	33 (66)	25 (54)	22 (68)	0	80 (52)
Corticosteroid dose (mg/j)	8 [0-26]	5 [0-19]	6 [2-8]	0	
Immunomodulator, n (%)	23 (46)	26 (57)	17 (53)	0	66 (43)

MSA, myositis specific antibody; MAA, myositis associated antibody; MMT8, manual muscle testing 8; MDAAT, myositis disease activity assessment tool; PGA, Physician global assessment; DM, dermatomyositis; ASyS, anti-synthetase syndrom; IMNM, immune mediated necrotizing myopathies; IBM, inclusion body myositis.

As expected, IBM patients were older and displayed a lower MMT8 score compared to DM and AsyS. MSA were detected in 70.6% of DM patients in the cohort 1 (anti-Mi2, n=10; -Tif1γ, n=12; -NXP2, n=7; -MDA5, n=5 and -SAE, n=2) AsyS were all positive (anti-Jo1, n=38; -PL7, n=4; -PL12, n=3 and –EJ, n=1), and all IMNM patients were seropositive (anti-SRP, n=13, -HMGCR, n=19). The disease duration was 301 [46-1411) days in DM, 857 [59-1935] in ASyS, 820 [196,8-2275] in IMNM and 1328 [646-2246] in IBM.

No difference was observed in the therapeutic profile, including the use of corticosteroids and immunosuppressors, between IMNM, DM, and AsyS while IBM patients did not receive any treatment.

### 3.2 Increased levels of type I and II IFNs depend on the myositis subgroups

**Figure 1:**
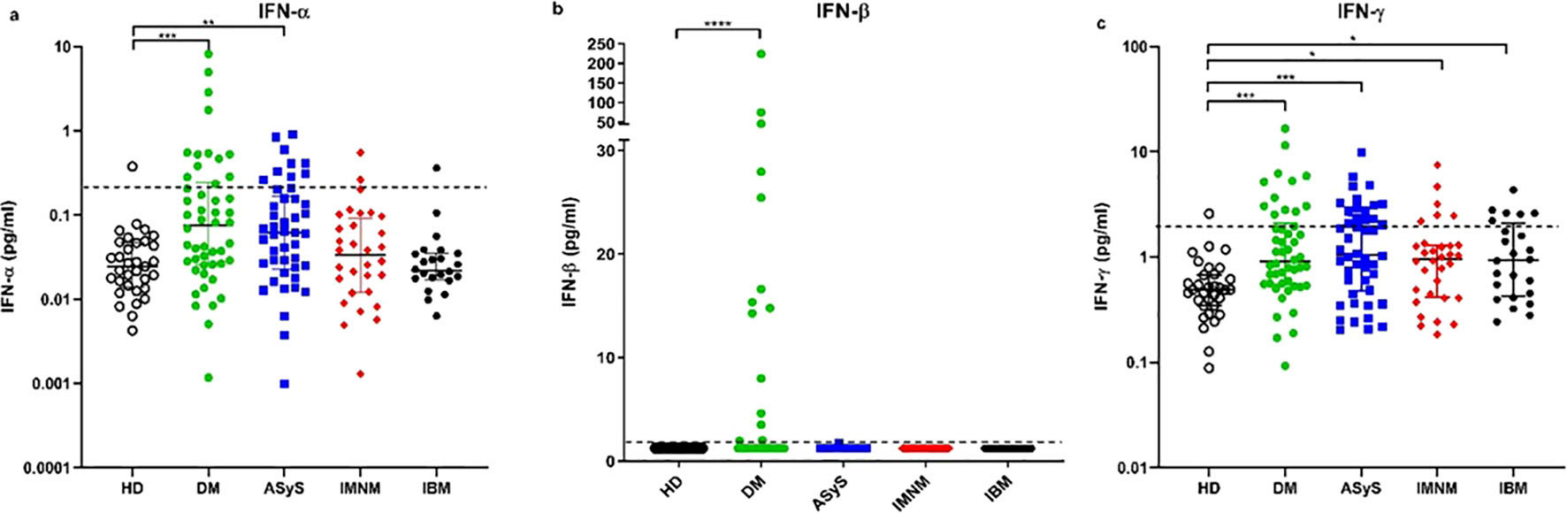
**(A)** Interferon alpha. **(B)** Interferon Beta. **(C)** Interferon gamma. IFN levels in IIM. DM, Dermatomyositis; ASyS, Anti-synthetase syndrome; IMNM, Immune-mediated necrotizing myopathies; IBM, Inclusion body myositis; HD, healthy donors - - mean ± 3 standard deviation or positivity threshold, *: p<0.05, ** p<0.005, ***: p<0.0005, **** p<0.00005.

Serum IFN-α level was significantly higher in DM (0.07 [0.03-0.23] pg/ml) and ASyS (0.07 [0.02-0.16] pg/ml) compared to HD (0.02 [0.01-0.05] pg/ml; p<0.005 and p<0.05 respectively) whereas it was not significantly different in IMNM (0.03 [0.01-0.09] pg/ml) or IBM (0.02 [0.02-0.03] pg/ml) compared to HD (**Figure 1A**).

Only DM patients had significantly higher IFN-β level (1.24 [1.24-6.31] pg/ml) compared to HD (1.24 [1.24-1.24] pg/ml, p<0.005) (**Figure 1B**).

IFN-γ level was significantly increased in all IIM subgroups (ASyS (1.05 [0.47-2.46] pg/ml), DM (0.90 [0.55-2.09] pg/ml), IMNM (0.96 [0.42-1.29] pg/ml) and IBM (0.93 [0.42-2.09] pg/ml)) compared with HD (0.46 [0.29-0.59] pg/ml), p<0.05) (**Figure 1C**).

### 3.3 IFN levels and disease activity

Correlation between IFNs levels and disease activity showed that PGA was strongly correlated with type-I IFNs, IFN-α (r=0.76 [0.60-0.86], p<0.001) and IFN-β (r=0.58 [0.35-0.74], p<0.01) in DM. A weak correlation with IFN-γ (r=0.36 [0.05-0.56], p=0.02) was observed. When we look at the correlations of the different IFN types with the core set measures assessing disease activity in DM, IFN-α correlates more with extra muscular domain (r=0.62 [0,47-0,73]) than muscle domain (CK: r= 0.29 [0,09-0,48] p<0.05 and MMT8: r= -0,31 [-0.48- -0.11] p<0.05). IFN-β correlates more with muscle domain (CK: r=0.45 [0.26-0.6] p<0.05 and MMT8: r=-0.34 [-0.51- -0.14] p<0.05) than extra muscular assessment (MDAAT: r=0.32 [0.12-0.50] p<0.05).

ASyS also demonstrated that PGA correlated with IFN-α (r=0.55 [0.34-0.76], p<0.001) and IFN-γ levels (r=0.46 [0.15-0.66], p=0.003). Of note, no ASyS patient presenting an active disease had increased IFN-β level. When we look at the correlations of the different IFN types with the core set measures, IFN-α correlates with extramuscular domain (r=0.34 [0,11-0,54]), CK (r= 0.45 [0,22-0,63] p<0.05 and MMT8: r= -0,27 [-0.49- -0.02] p<0.05). In ASyS patients, IFN-γ correlates more with muscle domain (CK: r=0.39 [0.08-0.63] p<0.05 and MMT8: r=-0.46 [-0.67- -0.17] p<0.05) than extra muscular assessment (r=0.31 [0.02-0.56] p<0.05).

In IMNM, only IFN-γ level was significantly correlated with disease activity (r=0.48 [0.14-0.71], p=0.006) whereas IFN-α (r=0.23 [-0.14-0.55], p=0.2) and IFN-β (r=-0.07 [-0.43-0.31], p=0.7) were not. IFN-γ correlates with CK levels (r=0.39 [0.08-0.63] p<0.05) but not with MMT8: r=-0.03 [-0.38- 0.33] p=0.87). Of note, correlation between CK levels and disease activity was very high (r=0.87 [0.73-0.94], p<0.001) for IMNM patients.

**Figure 2:**
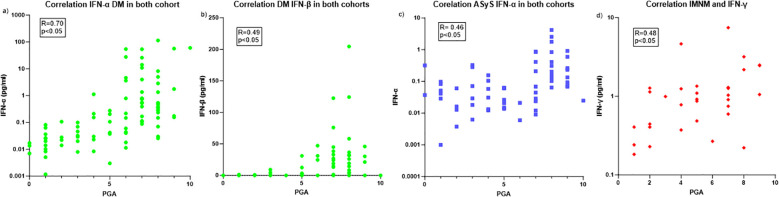
Correlation between IFNs and disease activity. **(A)** Correlation between IFN-α and DM disease activity. **(B)** Correlation between IFN-β and DM disease activity. **(C)** Correlation between IFN-α and ASyS disease activity. **(D)** Correlation between IFN-γ and IMNM disease activity. DM, Dermatomyositis; ASyS, Anti-synthetase syndrom; PGA, physician global assessment.

In IBM, IFN-γ correlates with CK (r=0.69 [0.35- 0.87] p<0.05) (**Figure 2D**) but not with MMT8.

Multivariate analysis including IFN-α, and IFN-γ showed that only IFN-α was associated with active disease in DM patients (OR=9.5 [3.1-45.9], p<0.001). Concerning ASyS patients, only IFN-α was statistically associated with disease activity (OR=5 [1.9-17.9], p=0.004), and there was a trend for IFN-γ (p=0.08). No IFN subtype was associated with disease activity in the IMNM subgroup.

### 3.4 Validation cohort

Given the correlation between type 1 IFN and disease activity in DM and ASyS, and that only IFN-γ correlate with IMNM disease activity whereas creatine kinase is a reliable biomarker in this condition, we focus on IFN type 1 in DM and ASyS. Thus, we build a second independent cohort to validate our observations in DM and ASyS.

Seventy patients were included (DM n=49 and ASyS n=21) in the validation cohort. Main patients’ characteristics are shown in **Table 2**. MSA were detected in 84% in the cohort 2 (anti-Mi2, n=6; -Tif1γ, n=12; -NXP2, n=7; -MDA5, n=12 and -SAE, n=2). ASyS were all but one antibody positive (anti-Jo1, n=12; -PL7, n=3; -PL12, n=2 -OJ n=1 and –EJ, n=2) Of note, in both cohort, 54 patients were naïve from treatment, including 28 DM patients, 21 ASyS patients and 6 IMNM patients.

**Table 2:** Patients characteristics in the second cohort at time of blood sampling.

Diagnosis	DM COHORT 2	ASyS cohort 2
n	49	21
Age (year)	49.2 ± 18.0	48.9 ± 17.9
MSA, n (%)	41 (84)	20 (95)
MAA, n (%)		
MMT8 (0-150)	140 [128-150]	142 [120-150]
CK level (UI/ml)	176 [65-958]	457 [159-4148]
MDAAT (0-60)	10 [5-15]	11 [6-15]
PGA (0-10)	6 [5-7]	7 [6-9]
Corticosteroids n (%)	26 (53)	9 (43)
Corticosteroids dose (mg/j)	5 (0-19]	0 [0-30]

MSA, myositis specific antibody; MAA, myositis associated antibody; MMT8, manual muscle testing 8; MDAAT, myositis disease activity assessment tool; PGA, Physician global assessment; DM, dermatomyositis; ASyS, anti-synthetase syndrom.

Correlation between IFN-α levels and disease activity in DM showed a good correlation (r=0.68 [0.49-0.81] p<0.05. Correlation with IFN-β was weak (r= 0.39 [0.11-0.61] p<0.05).

In ASyS, there was only a trend for the correlation between IFN-α and disease activity (r=0.37 [0.12-0.71] p=0.11).

When we pooled the two cohorts, DM patient (n=99) harboured a strong correlation between disease activity and type-I IFN, (IFN-α (r=0.70 [0.58-0.79], p<0.001) (**Figure 2A**) and IFN-β (r=0.49 [0.31-0.63], p<0.01) (**Figure 2B**)). ASyS also demonstrated that disease activity correlated with IFN-α in both cohort (n=67) (r=0.46 [0.23-0.63], p<0.001) (**Figure 2C**).

### 3.5 Sensitivity and specificity of IFNs to discriminate active and inactive DM and ASyS patients in both cohort

Next, we aimed to define the threshold level of IFN corresponding to active disease if there were a correlation between IFN and disease activity.

Active DM patients had higher level of IFN-α (0.49 [0.15-3.7] pg/ml) compared to non-active DM patients (0.03 [0.01-0.07] pg/ml, p<0.001) (**Supplementary Figure 1A**). ROC analysis showed an area under the curve (AUC) at 0.90 (IC95 0.84-0.96; p<0.001). For a threshold of 0.11 pg/ml, the sensitivity was 88% and the specificity 80% to discriminate active from non-active DM patients.

Active DM patients had higher level of IFN-β (4.58 [0.0-30.7]) compared to non-active DM patients (0 [0-0] pg/ml, p=0.0001) with an AUC at 0.71 (IC95 0.61-0.81; p<0.05) because of a lot of false negative. Of note, IFN-γ levels were higher in active DM patients (1.417 [0.81-2.74] pg/ml) compared to inactive ones (0.64 [0.38-1.20] pg/ml, p=0.007).

Active ASyS patients had higher IFN-α level (0.08 [0.03-0.38] pg/ml) compared to non-active ASyS patients (0.04 [0.01-0.09] pg/ml, p<0.001) with an AUC=0.69, IC95(0.56-0.82); p<0.05). Active ASyS patients had higher level of IFN-γ (2.02 [0.52-3.24] pg/ml) compared to non-active ASyS patients (0.86 [0.41-1.49] pg/ml, p<0.05) (**Supplementary Figure 2D**) and AUC=0.69, IC95(0.53-0.95); p<0.05.

Of note, for IMNM, active patients didn’t have higher level of IFN-γ (1.06 [0.75-2.18] pg/ml) than non-active patients (0.44 [0.31-1.19] pg/ml) p=0.06.

### 3.6 Different IFN secretion profiles depending on IIMs subgroups and myositis specific antibodies

**Figure 3:**
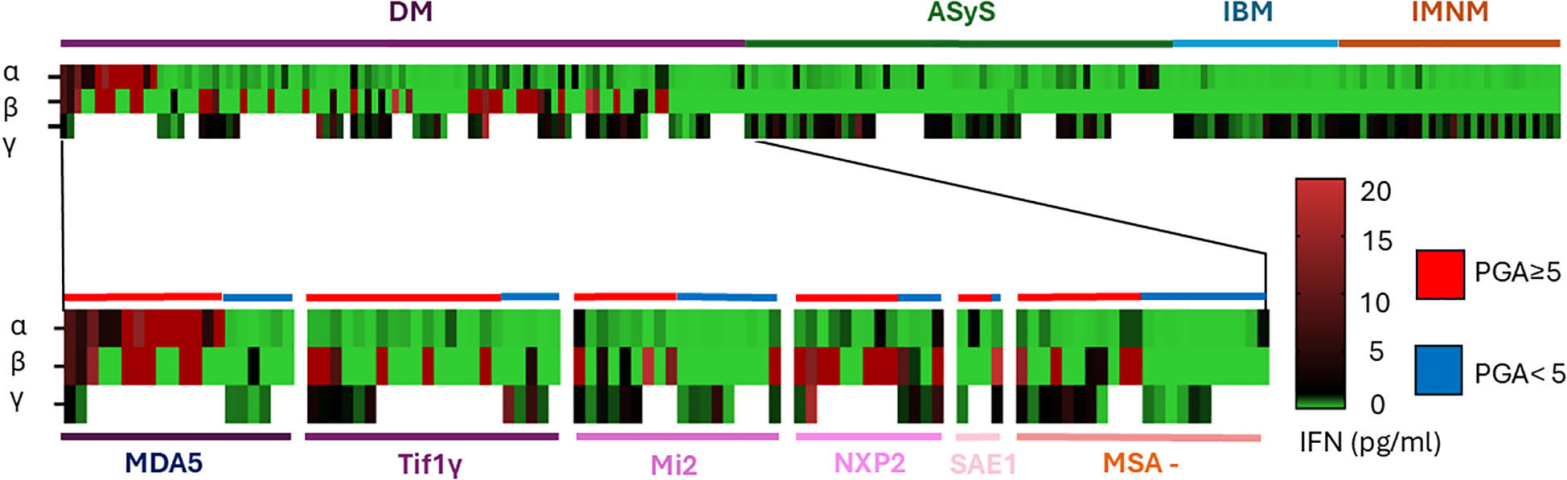
Different cytokine profile according to subgroup of myositis and antibody in DM. Each rows represent a patient, each colomn an IFN subtype and the color define the quantity of IFN secretion in pg/ml.

The levels of IFN-α, IFN-β, and IFN-γ for each patient are presented in **Figure 3**, where all tested patients are grouped by IIMs subtypes and according to disease activity.

In DM, patients with anti-MDA5 antibodies had higher levels of IFN-α than the others patients (6.58 [0.12-47.16] pg/ml vs 0.14 [0.02-0.22] pg/ml p<0.005) (**Figure 3** and **Supplementary Figure 2A**). Anti-NXP2 positive antibody patients had higher IFN-β level (25.6 [0.45-67.03] pg/ml) than patients with Tif1γ antibodies (0.0 [0.0-7.3] pg/ml p<0.05) but not again the other ASM (**Figure 3** and **Supplementary Figure 2**). Focusing on naïve patients (n=28/99), 23 patients had an increased level of IFN-α above the 2 SD threshold (0.22 pg/ml), 18 for IFN-β (2.50 pg/ml) and only 2 for IFN-γ.

## 4 Discussion

In this study, using two independent cohorts, we demonstrated that type I IFN, particularly IFN-α, are reliable biomarkers of disease activity in patients with DM and ASyS. While IFN-α levels were elevated in both conditions, IFN-β was only significantly increased in DM patients. Furthermore, IFN-α showed a strong association with the MDA5-positive subgroup and exhibited superior performance as a biomarker for disease activity in DM. Type II interferon (IFN-γ) was elevated across all myositis subgroups, with the strongest correlation observed in ASyS, suggesting its relevance as a disease activity biomarker in this cohort.

The concept of an interferon signature has been previously documented in DM, with studies identifying the expression of IFN-stimulated genes in muscle, skin, and blood samples (26–28). The IFN score, which combines the expression of multiple IFN-stimulated genes, has been used to assess disease activity, particularly in DM. While it serves as a valuable tool, it is not yet standardized for routine clinical use and requires RNA extraction (7).

Moreover, although prior research has shown that the IFN score correlates with cutaneous disease activity in DM (11), our study extends these findings by linking serum IFN levels with overall disease activity, rather than focusing solely on skin involvement. Only one previous study employed Digital ELISA to measure IFN-α blood levels in adult DM (11), noting the strong correlation between IFN-α and the IFN gene signature (11, 24). This earlier study demonstrated a significant correlation between IFN-β levels and cutaneous disease activity, but did not evaluate overall disease activity. Additionally, this technology has been successfully applied to other autoimmune diseases, such as lupus, where it showed a correlation with disease activity (25).

Historically, IFN-β has been considered the most reliable biomarker for DM activity (29). However, our study reveals that both IFN-α and IFN-β are reliable biomarkers in DM, with IFN-β being more specific to DM, whereas IFN-α appears to have broader relevance across various myositis subgroups.

Notably, we observed that some DM patients, particularly those with anti-MDA5 or anti-SAE antibodies, had elevated levels of IFN-α but not IFN-β. This finding suggests that the IFN pathway may vary depending on the DM subgroup, with significant therapeutic implications. This variability is particularly important, as therapeutic monoclonal antibodies may block only the IFN-α or IFN-β pathways.It is important to note that the detection limit for IFN-α was 300 times lower than that for IFN-β. This suggests that the utility of monitoring IFN-β levels could increase as the detection limit improves. Currently, no commercially available assays for IFN-β have been developed by the manufacturer.

We also observed that IFN-γ, a type II interferon, was elevated in all myositis subgroups, with the strongest correlation with disease activity observed in ASyS. This finding is consistent with previous studies indicating that patients with anti-Jo1 antibodies exhibit an IFN signature and supports the notion that ASyS may be considered a type II interferonopathy (30). This distinction underscores the different underlying immune pathways in these conditions: DM is characterized by a type I interferon signature (IFN-α and IFN-β), whereas ASyS is predominantly associated with a type II interferon signature. Muscle tissue analysis has shown that DM patients express IFN-related proteins (31, 32), while ASyS patients predominantly overexpress MHC-II, a protein induced by type II IFN (33). These findings highlight the need for tailored therapeutic strategies, as the IFN pathways involved in these diseases differ.

In the case of IMNM, only IFN-γ levels correlated with disease activity, with CK levels being a better biomarker, as previously shown (4). IFN-γ levels may reflect a involvement of Th1 immune responses such as CD8+ T cells, which play a key role in the pathophysiology of IBM and ASyS (12, 13) but these cells are sparse or absent in IMNM. On the other hand, macrophages, which are the predominant immune cells infiltrating IMNM muscle tissue, may contribute to the increased levels of IFN-γ (4). These findings further emphasize the distinct immune mechanisms driving disease activity in the different subtypes of myositis.

A key limitation of this study is the absence of a universally accepted gold standard for assessing disease activity in myositis. Both DM and ASyS are multisystemic diseases, making the measurement of disease activity challenging. In the absence of specific biomarkers, disease activity is typically assessed using a combination of clinical, radiological, and functional criteria. However, many of these methods, particularly in non-muscular disease domains (e.g., skin, lungs, joints), rely on subjective clinical evaluation or imaging techniques that may not directly measure inflammatory disease activity or could be influenced by sequelae of previous diseases. The lack of objective biomarkers for these disease domains complicates the overall assessment of disease activity. To address this, the ACR/EULAR has developed composite improvement scores to assess disease activity based on changes over time (3).

While IFN levels may not yet fully replace clinical tools, our study suggests that monitoring IFN levels could be more sensitive in assessing disease activity than traditional clinical measures. Further validation in larger, independent cohorts, as well as longitudinal studies, is needed to establish IFN-α and IFN-γ as routine biomarkers for disease monitoring and to refine therapeutic strategies, particularly in DM.

## 5 Conclusion

In conclusion, this study supports the role of type I IFN (IFN-α and IFN-β) and type II IFN (IFN-γ) as reliable biomarkers for disease activity in DM and ASyS. Serum IFN-α levels, measured using SIMOA technology, correlate closely with clinical disease activity in DM, while IFN-γ could be a useful biomarker in ASyS. The findings also suggest that myositis-specific antibodies are associated with distinct IFN secretion profiles, which may help guide personalized treatment strategies. These results should be confirmed in independent prospective studies to validate the clinical utility of IFN biomarkers and optimize therapeutic approaches, especially in DM.

## Data Availability

The original contributions presented in the study are included in the article/**Supplementary Material**. Further inquiries can be directed to the corresponding author.

## References

[1] Mariampillai K Granger B Amelin D Guiguet M Hachulla E Maurier F. Development of a new classification system for idiopathic inflammatory myopathies based on clinical manifestations and myositis-specific autoantibodies. JAMA Neurol. (2018) 75:1528−37. doi: 10.1001/jamaneurol.2018.2598 30208379 PMC6583199

[2] Selva-O’Callaghan A Pinal-Fernandez I Trallero-Araguás E Milisenda JC Grau-Junyent JM Mammen AL. Classification and management of adult inflammatory myopathies. Lancet Neurol. (2018) 17:816−28. doi: 10.1016/S1474-4422(18)30254-0 30129477 PMC11646336

[3] Aggarwal R Rider LG Ruperto N Bayat N Erman B Feldman BM. 2016 American College of Rheumatology/European League Against Rheumatism criteria for minimal, moderate, and major clinical response in adult dermatomyositis and polymyositis: An International Myositis Assessment and Clinical Studies Group/Paediatric Rheumatology International Trials Organisation Collaborative Initiative. Ann Rheum Dis mai. (2017) 76:792−801. doi: 10.1002/art.40064 PMC5496443 28385805

[4] Allenbach Y Arouche-Delaperche L Preusse C Radbruch H Butler-Browne G Champtiaux N. Necrosis in anti-SRP+ and anti-HMGCR+myopathies: Role of autoantibodies and complement. Neurology. (2018) 90:e507−17. doi: 10.1212/WNL.0000000000004923 29330311

[5] Mathur T Manadan AM Thiagarajan S Hota B Block JA. The utility of serum aldolase in normal creatine kinase dermatomyositis. J Clin Rheumatol. (2014) 20:47−8. doi: 10.1097/RHU.0000000000000062 24356484

[6] Bolko L Jiang W Tawara N Landon-Cardinal O Anquetil C Benveniste O. The role of interferons type I, II and III in myositis: A review. Brain Pathol. (2021) 31:e12955. doi: 10.1111/bpa.12955 34043262 PMC8412069

[7] Greenberg SA Pinkus JL Pinkus GS Burleson T Sanoudou D Tawil R. Interferon-alpha/beta-mediated innate immune mechanisms in dermatomyositis. Ann Neurol. (2005) 57:664−78. doi: 10.1002/ana.20464 15852401

[8] Greenberg SA Higgs BW Morehouse C Walsh RJ Kong SW Brohawn P. Relationship between disease activity and type 1 interferon- and other cytokine-inducible gene expression in blood in dermatomyositis and polymyositis. Genes Immun. (2012) 13:207−13. doi: 10.1038/gene.2011.61 21881594

[9] Baechler EC Bauer JW Slattery CA Ortmann WA Espe KJ Novitzke J. An interferon signature in the peripheral blood of dermatomyositis patients is associated with disease activity. Mol Med. (2007) 13:59−68. doi: 10.2119/2006-00085.Baechler 17515957 PMC1869622

[10] Wong D Kea B Pesich R Higgs BW Zhu W Brown P. Interferon and biologic signatures in dermatomyositis skin: specificity and heterogeneity across diseases. PLoS One. (2012) 7:e29161. doi: 10.1371/journal.pone.0029161 22235269 PMC3250414

[11] Huard C Gullà SV Bennett DV Coyle AJ Vleugels RA Greenberg SA. Correlation of cutaneous disease activity with type 1 interferon gene signature and interferon β in dermatomyositis. Br J Dermatol. (2017) 176:1224−30. doi: 10.1111/bjd.15006 27564228

[12] Allenbach Y Chaara W Rosenzwajg M Six A Prevel N Mingozzi F. Th1 response and systemic treg deficiency in inclusion body myositis. PLoS One. (2014) 9:e88788. doi: 10.1371/journal.pone.0088788 24594700 PMC3942319

[13] Hervier B Perez M Allenbach Y Devilliers H Cohen F Uzunhan Y. Involvement of NK cells and NKp30 pathway in antisynthetase syndrome. J Immunol. (2016) 197:1621−30. doi: 10.4049/jimmunol.1501902 27511738

[14] Pinal-Fernandez I Casal-Dominguez M Derfoul A Pak K Plotz P Miller FW. Identification of distinctive interferon gene signatures in different types of myositis. Neurology. (2019) 93:e1193−204. doi: 10.1212/WNL.0000000000008128 31434690 PMC6808530

[15] Rigolet M Hou C Baba Amer Y Aouizerate J Periou B Gherardi RK. Distinct interferon signatures stratify inflammatory and dysimmune myopathies. RMD Open. (2019) 5:e000811. doi: 10.1136/rmdopen-2018-000811 30886734 PMC6397431

[16] Tabata MM Hodgkinson LM Wu TT Li S Huard C Zhao S. The type I interferon signature reflects multiple phenotypic and activity measures in dermatomyositis. Arthritis Rheumatol. (2023) 75:1842−9. doi: 10.1002/art.v75.10 37096447

[17] Wilson DH Rissin DM Kan CW Fournier DR Piech T Campbell TG. The simoa HD-1 analyzer: A novel fully automated digital immunoassay analyzer with single-molecule sensitivity and multiplexing. J Lab Autom. (2016) 21:533−47. doi: 10.1177/2211068215589580 26077162

[18] Lundberg IE Tjärnlund A Bottai M Werth VP Pilkington C de Visser M. 2017 European league against rheumatism/american college of rheumatology classification criteria for adult and juvenile idiopathic inflammatory myopathies and their major subgroups. Arthritis Rheumatol (Hoboken NJ). (2017) 69:2271−82. doi: 10.1002/art.40320 PMC5846474 29106061

[19] Lloyd TE Mammen AL Amato AA Weiss MD Needham M Greenberg SA. Evaluation and construction of diagnostic criteria for inclusion body myositis. Neurology. (2014) 83:426−33. doi: 10.1212/WNL.0000000000000642 24975859 PMC4132572

[20] Allenbach Y Mammen AL Benveniste O Stenzel W. Immune-Mediated Necrotizing Myopathies Working Group. 224th ENMC International Workshop:: Clinico-sero-pathological classification of immune-mediated necrotizing myopathies Zandvoort, The Netherlands, 14-16 October 2016. Neuromuscul Disord. (2018) 28:87−99. doi: 10.1016/j.nmd.2017.09.016 29221629

[21] Stenzel W Mammen AL Gallay L Holzer MT Kleefeld F Benveniste O. 273rd ENMC International workshop: Clinico-Sero-morphological classification of the Antisynthetase syndrome. Amsterdam, The Netherlands, 27-29 October 2023. Neuromuscul Disord. (2024) 45:104453. doi: 10.1016/j.nmd.2024.104453 39490006

[22] Mammen AL Allenbach Y Stenzel W Benveniste O. ENMC 239th workshop study group. 239th ENMC international workshop: classification of dermatomyositis, amsterdam, the Netherlands, 14-16 december 2018. Neuromuscul Disord. (2020) 30:70−92. doi: 10.1016/j.nmd.2019.10.005 31791867

[23] Flammer JR Dobrovolna J Kennedy MA Chinenov Y Glass CK Ivashkiv LB. The type I interferon signaling pathway is a target for glucocorticoid inhibition. Mol Cell Biol. (2010) 30:4564−74. doi: 10.1128/MCB.00146-10 20679482 PMC2950533

[24] Rodero MP Decalf J Bondet V Hunt D Rice GI Werneke S. Detection of interferon alpha protein reveals differential levels and cellular sources in disease. J Exp Med. (2017) 214:1547−55. doi: 10.1084/jem.20161451 28420733 PMC5413335

[25] Mathian A Mouries-Martin S Dorgham K Devilliers H Barnabei L Ben Salah E. Monitoring disease activity in systemic lupus erythematosus with single-molecule array digital ELISA quantification of serum interferon-α. Arthritis Rheumatol (Hoboken NJ). (2019) 71(5):756–65. doi: 10.1002/art.40792 30507062

[26] Allenbach Y Leroux G Suárez-Calvet X Preusse C Gallardo E Hervier B. Dermatomyositis with or without anti-melanoma differentiation-associated gene 5 antibodies: common interferon signature but distinct NOS2 expression. Am J Pathol. (2016) 186:691−700. doi: 10.1016/j.ajpath.2015.11.010 26806087

[27] Salajegheh M Kong SW Pinkus JL Walsh RJ Liao A Nazareno R. Interferon-stimulated gene 15 (ISG15) conjugates proteins in dermatomyositis muscle with perifascicular atrophy. Ann Neurol. (2010) 67:53−63. doi: 10.1002/ana.21805 20186858 PMC2875060

[28] Rice GI Forte GMA Szynkiewicz M Chase DS Aeby A Abdel-Hamid MS. Assessment of interferon-related biomarkers in Aicardi-Goutières syndrome associated with mutations in TREX1, RNASEH2A, RNASEH2B, RNASEH2C, SAMHD1, and ADAR: a case-control study. Lancet Neurol. (2013) 12:1159−69. doi: 10.1016/S1474-4422(13)70258-8 24183309 PMC4349523

[29] Liao AP Salajegheh M Nazareno R Kagan JC Jubin RG Greenberg SA. Interferon β is associated with type 1 interferon-inducible gene expression in dermatomyositis. Ann Rheum Dis. (2011) 70:831−6. doi: 10.1136/ard.2010.139949 21177291

[30] Ekholm L Vosslamber S Tjärnlund A de Jong TD Betteridge Z McHugh N. Autoantibody specificities and type I interferon pathway activation in idiopathic inflammatory myopathies. Scand J Immunol. (2016) 84:100−9. doi: 10.1111/sji.2016.84.issue-2 27173897

[31] Uruha A Allenbach Y Charuel JL Musset L Aussy A Boyer O. Diagnostic potential of sarcoplasmic MxA expression in subsets of dermatomyositis. Neuropathol Appl Neurobiol. (2019) 45(5):513–22. doi: 10.1111/nan.12519 30267437

[32] Uruha A Nishikawa A Tsuburaya RS Hamanaka K Kuwana M Watanabe Y. Sarcoplasmic MxA expression: A valuable marker of dermatomyositis. Neurology. (2017) 88:493−500. doi: 10.1212/WNL.0000000000003568 28039312

[33] Aouizerate J De Antonio M Bassez G Gherardi RK Berenbaum F Guillevin L. Myofiber HLA-DR expression is a distinctive biomarker for antisynthetase-associated myopathy. Acta Neuropathol Commun. (2014) 2:154. doi: 10.1186/s40478-014-0154-2 25339355 PMC4210467

