# Supplementary figures and images for: Ultrasensitive interferons quantification reveals different cytokine profile secretion in inflammatory myopathies and can serve as biomarkers of activity in dermatomyositis
Source: Front Immunol. 2025 Feb 12;16:1529582. doi: 10.3389/fimmu.2025.1529582 (PMC11861187; doi:10.3389/fimmu.2025.1529582)

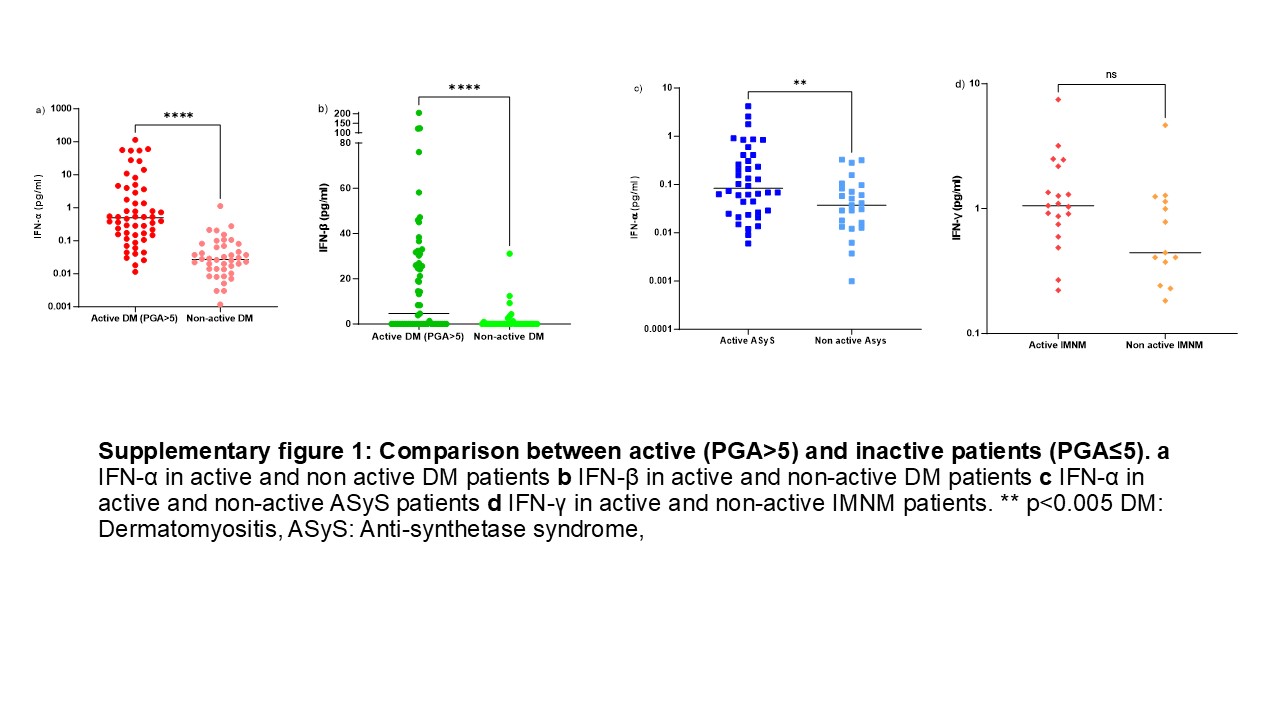

Supplement: Supplementary file 1 [file Image1.jpeg]

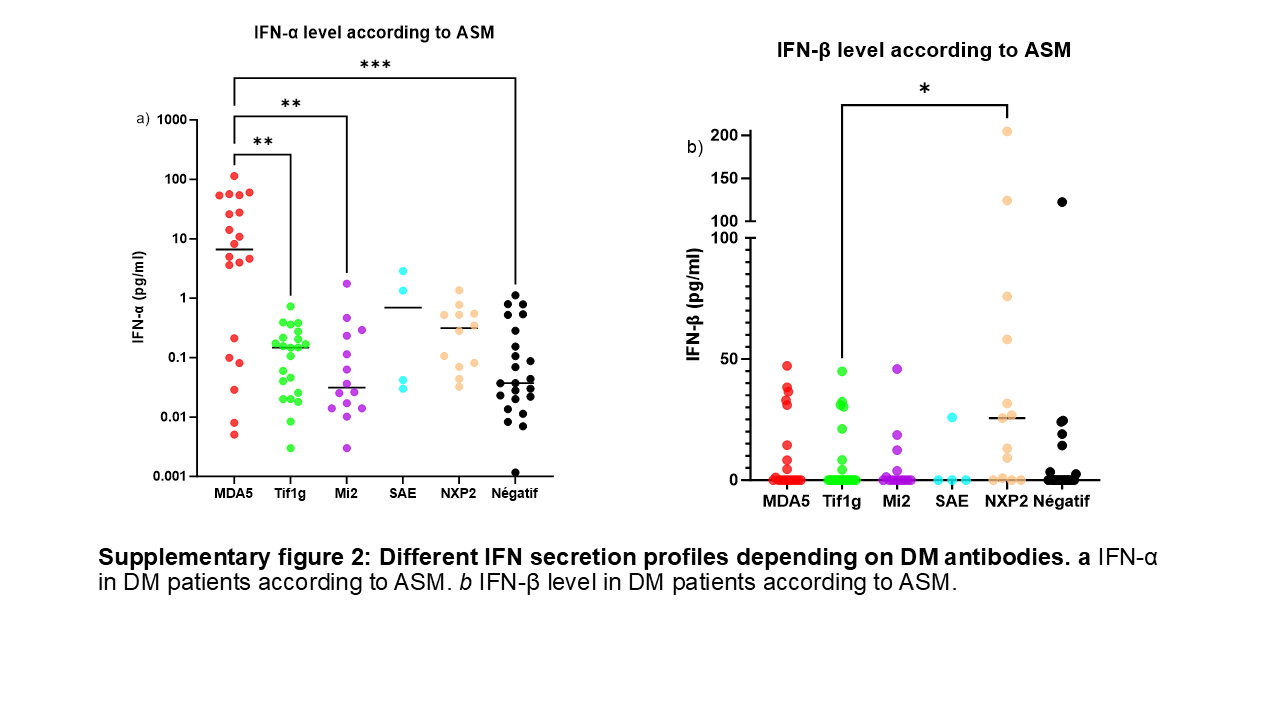

Supplement: Supplementary file 2 [file Image2.tif]
